# Establishing a Pre‐Vocational Surgical Society: Early Experience, Outputs and Educational Value

**DOI:** 10.1111/ans.70846

**Published:** 2026-07-08

**Authors:** Jack Gerrard, Teresa Liew, Alexandra Stathis

**Affiliations:** ^1^ Pre‐Vocational Australasian Surgical Society Melbourne Victoria Australia; ^2^ Department of Surgery Peter MacCallum Cancer Centre Melbourne Victoria Australia; ^3^ Department of Surgery Princess Alexandra Hospital Woolloongabba Queensland Australia; ^4^ Department of Surgery Port Macquarie Base Hospital Port Macquarie New South Wales Australia; ^5^ School of Medicine University of Notre Dame Australia Darlinghurst New South Wales Australia

**Keywords:** education, financial stress, internship and residency, mentoring, surgeons

## 1 Introduction

Developing a skilled future surgical workforce requires engagement across the continuum of medical training. The prevocational period includes work after completion of basic medical qualifications before entry into a formal training pathway, such as the Royal Australasian College of Surgeons (RACS) Surgical Education and Training (SET) program. While medical student surgical societies may foster early interest in surgery, structured engagement after medical school until gaining entry into surgical training remains variable [1]. In Australia and Aotearoa New Zealand, the transition from medical graduate to junior doctor represents a critical phase where clinicians consolidate clinical skills, refine career intentions and begin preparing for competitive specialty training [2, 3, 4]. However, targeted surgical education, mentorship and professional development outside clinical rotations is often limited [3, 4, 5, 6].

In response, the Pre‐Vocational Australasian Surgical Society (PVASS) was established to support doctors interested in surgery across Australia and Aotearoa New Zealand. This article outlines the rationale for its inception, reviews early activities and considers its educational value within surgical training and governance frameworks.

## 2 Rationale

Prevocational doctors encounter inconsistent surgical exposure, which is often service‐oriented, with variable support and access to protected teaching time [1, 5, 6, 7, 8].

Additionally, prevocational doctors in Australia and Aotearoa New Zealand are spending more years progressing through internship, residency and unaccredited registrar roles before commencing surgical training [7, 8, 9, 10, 11]. Preparation for surgical training remains costly with estimates reporting successful candidates may spend over $50 000 AUD on examinations, courses and professional development [12, 13].

A prevocational society addresses the need for sustained mentorship, career guidance and equitable access to educational opportunities and professional development, through providing a collective voice for prevocational doctors navigating this prolonged, uncertain and increasingly complex transition into specialty training [10, 14, 15].

## 3 Development and Governance

PVASS was founded in 2021 with early engagement from surgical trainees, consultants and RACS. The society's founding objectives were to: provide accessible surgical education to prevocational doctors; facilitate mentorship and networking; support early career development, including research and training preparation; and advocate for prevocational doctors within RACS frameworks and hospital networks.

Since its inception, PVASS has become a registered charity with the Australian Charities and Not‐for‐profits Commission. The organisation has benefited from the contributions of 44 volunteers, reflecting broad engagement and sustainability. Governance includes an executive committee that reports to a board of directors, who oversee strategy, compliance and ongoing activity.

## 4 Activities and Outputs

PVASS has delivered a range of educational and professional development activities, predominantly through videoconference, enabling broad geographic reach across Australia and Aotearoa New Zealand. Events have included surgical specialty information sessions, examination and interview preparation, career development, advocacy and academic seminars introducing research participation. Average event attendance has been approximately 80 participants, demonstrating interest and engagement. Additionally, PVASS produces educational factsheets of surgical conditions relevant to prevocational practice. The society has a social media presence of over 1200 followers and a secure online group where more than 400 registered doctors can ask for advice or guidance.

## 5 Mentoring

PVASS is currently introducing a structured mentoring initiative that pairs junior doctors with SET trainees and consultants, offering longitudinal advice and support for career planning, portfolio development, examination preparation and work–life considerations. Misconceptions may exist regarding the personality and lifestyle of surgeons, and exposure to mentors from a variety of backgrounds may inspire the most talented prevocational doctors to pursue a surgical career [16, 17]. Furthermore, mentorship can positively influence career preparedness, satisfaction and retention within surgical training [10, 14, 15].

## 6 Advocacy

PVASS has maintained a working relationship with RACS since its inception, enabling meaningful representation of prevocational perspectives within surgical education and governance structures. As part of this, PVASS secured mandated representation on the Prevocational Skills and Education Committee (PSEC). This representation includes discussion of issues affecting prevocational doctors, presentations at RACS strategy meetings and advocacy regarding the impact of selection requirements for SET or costs of RACS initiatives.

In 2023, PVASS also participated in a RACS Managing Bias Working Party, contributing the voice of prevocational doctors from marginalised or minority backgrounds.

Prevocational doctors invest significant time and financial resources into research, higher education, or additional training over a prolonged period, sometimes while balancing family responsibilities. Changes to selection criteria or points allocation—commonly perceived as ‘changing goalposts’—may disproportionately disadvantage these doctors [5, 10, 18]. PVASS has highlighted these impacts and endeavoured to increase transparency with advance notice of selection changes. In recent years, several specialty training selection boards have moved toward hurdle‐based selection models or improved communication regarding future requirements. Ongoing PSEC representation ensures that prevocational perspectives are considered prior to policy decisions, mitigating unintended consequences and financial strain.

## 7 Perceived Educational Value

Informal feedback suggests that PVASS activities address unmet educational and professional needs. Attendees report improved understanding of surgical pathways, increased confidence in navigating requirements and greater awareness of mentorship and research opportunities.

The society has 601 members across multiple levels of training with varied specialty interests (Tables 1 and 2). Equity of access is an important consideration in surgical training. The online delivery of all events at no cost enhances accessibility and helps mitigate disparities in opportunity for doctors in smaller hospitals or regional settings (Table 3) [9, 18]. Events are designed to complement hospital‐based education and tailored to the unique challenges of the prevocational period.

**TABLE 1:** Summary of specialty interests of PVASS members.

Specialty of interest	Members
Undecided	107
General Surgery	143
Cardiothoracic Surgery	19
Otolaryngology (ENT)	34
Plastic & Reconstructive Surgery (PRS)	44
Neurosurgery	31
Urology	27
Vascular Surgery	16
Orthopaedic Surgery	60
Ophthalmology	4
Oral and Maxillofacial Surgery (OMFS)	4
Paediatric surgery	7
Data not available^a^	105
Total	601

*Note:* The number of members in each category is presented directly in each row.

^a^ These members did not disclose their specialty of interest upon registration.

**TABLE 2:** Level of training of PVASS members when joining as members.

Career stage when joining	Members
Medical student	299
Intern	155
PGY2	52
PGY3	19
PGY4	20
PGY5	8
PGY6	4
PGY7	2
PGY8	2
PGY9+	4
Data not available^a^	36
Total	601

*Note:* The number of members in each category is presented directly in each row. Please note that the distribution of level of training of PVASS members today would reflect a more senior group of prevocational doctors, considering some members may have joined 5 years ago when the society was established.

^a^ These members did not disclose their career stage upon registration.

**TABLE 3:** Geographical distribution of PVASS members.

Geographical location	Members
Australian Capital Territory	7
New South Wales	112
Aotearoa New Zealand	35
Northern Territory	1
Papua New Guinea	1
Queensland	141
South Australia	26
Tasmania	13
Victoria	220
Western Australia	29
Data not available^a^	16
Total	601

*Note:* The number of members in each category is presented directly in each row.

^a^ These members did not disclose their location upon registration.

## 8 Limitations

This review is descriptive and relies on participation metrics and informal feedback. Objective evaluation of educational outcomes with validated confidence measures or longitudinal tracking of career progression is not yet available. Attendance figures may not fully capture deeper engagement or long‐term benefit.

## 9 Future Directions

Future priorities include formal evaluation of mentoring programmes, expansion of inter‐hospital collaboration and structured assessment of educational outcomes.

## 10 Conclusion

The establishment of a pre‐vocational surgical society is feasible and appropriate with the continued support of volunteers and surgical governance bodies. Early experience demonstrates educational, mentorship and advocacy benefits for junior doctors considering surgical careers. Through structured events, mentoring and engagement with surgical governance bodies, PVASS represents a valuable adjunct to existing surgical training frameworks and supports a more informed, equitable and prepared future surgical workforce.

## Author Contributions

**Teresa Liew:** writing – review and editing. **Jack Gerrard:** conceptualization, methodology, data curation, validation, investigation, writing – original draft, writing – review and editing, project administration. **Alexandra Stathis:** writing – review and editing.

## Funding

The authors have nothing to report.

## Conflicts of Interest

The authors declare no conflicts of interest.

## Data Availability

The data that support the findings of this study are available from the corresponding author upon reasonable request.
